# Optimizing document management and retrieval with multimodal transformers and knowledge graphs

**DOI:** 10.1371/journal.pone.0323966

**Published:** 2025-06-11

**Authors:** Yali Chen, Bin Hu, Yajuan Liu

**Affiliations:** 1 Archives of Nantong University, Nantong, China; 2 School of Artificial Intelligence and Computer Science, Nantong University, Nantong, China; 3 Archives of Shandong Agricultural University, Taian, China; Philadelphia University, JORDAN

## Abstract

In the digital age, multimodal archival data is experiencing explosive growth, and how to efficiently and accurately retrieve information from it has become a key challenge. Traditional retrieval methods struggle to effectively handle multi-source heterogeneous multimodal data, leading to poor retrieval accuracy and efficiency. To address this issue, this paper proposes the MDKG-RL model, which organically integrates knowledge graph reasoning, deep reinforcement learning dynamic optimization, and multimodal Transformer architecture to achieve deep semantic understanding of multimodal data and intelligent optimization of retrieval strategies. The experiments, based on the ICDAR 2023 and AIDA Corpus datasets, show that MDKG-RL achieves a mean reciprocal rank (MRR) of 0.85, a normalized discounted cumulative gain (NDCG) of 0.88, and an entity linking accuracy of 92.4%. Compared to the baseline model, MRR improves by 13.3%, NDCG increases by 12.8%, and response time is reduced by 38.2%, significantly outperforming other comparison models. Ablation experiments also confirm the indispensability of each module. Visual analysis further demonstrates the model’s clear advantages in retrieval accuracy and efficiency, though error analysis reveals its shortcomings in handling long-tail entities and cross-modal ambiguity. The MDKG-RL model provides an innovative and effective solution for multimodal archival retrieval, not only improving retrieval performance but also laying the foundation for future research. In the future, model performance and generalization capabilities can be further enhanced by expanding data, optimizing strategies, and extending application scenarios, thereby promoting the development and application of multimodal retrieval technology in the fields of information management and knowledge discovery.

## Introduction

In the wake of the digital revolution, the field of archival management is undergoing profound changes. The global pace of archival digitization has accelerated, with the International Council on Archives (ICA) statistics from 2024 showing that the global institutional-level archival digitization rate has risen to 82%. However, the effective utilization rate of archival information remains around 35% [1]. This stark contrast highlights the significant shortcomings of traditional archival management models in dealing with the vast, complex, and dynamically changing archival resources [2].

The traditional keyword-based archival management approach faces numerous challenges in multimodal data processing, semantic understanding, and dynamic updates. Multimodal archival data (such as text, images, audio, etc.) has significant heterogeneity, which makes it difficult to integrate and forms isolated “information silos” [3]. For instance, textual archives focus on semantic expression, while image archives emphasize visual features, making it difficult to process and retrieve them using a unified approach [4]. Moreover, the complex semantic relationships embedded in archival content, such as the connections between people, events, and time, far exceed the capabilities of simple keyword matching [5]. Additionally, as archival data is constantly updated, traditional static ontology models are unable to adapt to such dynamic changes and cannot effectively organize and manage new archival information in a timely manner [6]. These problems lead directly to low recall and poor precision in archival retrieval, severely limiting the full potential of archival resources.

In recent years, the technology of knowledge graphs, with its powerful semantic modeling capabilities, has brought new ideas and methods to archival management. Numerous studies have attempted to apply knowledge graphs in archival management, aiming to achieve semantic relationships between archival entities by constructing domain-specific knowledge graphs. However, current research still exhibits significant technical shortcomings [7]. On one hand, multimodal data fusion often only involves simple feature concatenation and does not achieve true deep semantic alignment; on the other hand, the construction of knowledge graphs often relies on manual annotation, which is inefficient and unable to meet the dynamic update needs of archival data [8]. Furthermore, the optimization of retrieval strategies lacks an effective user feedback mechanism, making it difficult to personalize adjustments based on actual user needs and usage habits. For example, Ma *et al*. (2023) proposed an archival knowledge graph that performs poorly when handling the evolution of entity relationships in new archival data, relying only on static relationship reasoning [9]; Liao *et al*. (2024) developed a multimodal retrieval model that achieves an F1 score of only 0.78 in the image-text alignment task, which is still far from the expected performance in practical applications [10].

Advanced multimodal models such as CLIP and UNITER have made significant progress in semantic alignment and cross-modal tasks, especially in vision-language tasks. CLIP demonstrates excellent performance on vision-language tasks through large-scale image-text contrastive learning, while UNITER improves the accuracy of semantic understanding through multimodal pre-training models. However, these existing methods mainly focus on aligning multimodal features through self-supervised learning, but still have shortcomings in handling complex semantic relationships, dynamic data updates, and personalized retrieval. Pre-trained models (such as BERT) have made significant progress in text semantic understanding, but they face difficulties in effectively integrating multimodal data due to differences in feature spaces across modalities [11]. Graph Neural Networks (GNNs) have unique advantages in modeling entity relationships, but traditional GNN models encounter bottlenecks in computational efficiency when dealing with dynamic graph updates [12]. Reinforcement Learning (RL) has shown powerful strategy optimization capabilities in recommendation systems, but its application in archival retrieval is still in the exploratory stage [13].

To address these issues, this paper proposes a Multi-modal Dynamic Knowledge Graph with Reinforcement Learning for Archival Retrieval (MDKG-RL) model. This model integrates Transformer, Graph Neural Networks, and Deep Reinforcement Learning technologies. It uses cross-modal Transformer to achieve unified semantic representation of multimodal archival data, builds dynamic knowledge graphs using Graph Neural Networks, and optimizes retrieval strategies through Deep Reinforcement Learning, aiming to overcome the technological bottlenecks in traditional archival management.

The main contributions of this paper are as follows:

- Design of a multimodal Transformer fusion framework to achieve deep semantic alignment of text, image, and audio features.
- Proposal of a dynamic knowledge graph updating mechanism that supports incremental learning for automatic expansion of entity relationships.
- Construction of a user feedback-driven reinforcement learning model to achieve adaptive optimization of retrieval strategies.

The structure of this paper is as follows: the Related Works section reviews existing literature and methodologies relevant to our research; the MDKG-RL Model Construction section presents the detailed design and implementation of the proposed model; the Experiment section provides an evaluation of the model’s performance through empirical tests; and the Conclusion section summarizes the findings and suggests potential directions for future research.

## Related works

### Application of knowledge graphs in archival management

As a semantic network technology, knowledge graphs have brought innovative ideas to archival management. They can effectively represent and explore the complex relationships between archival entities, enhancing the relevance and comprehensibility of archival information. Early archival knowledge graph construction relied heavily on traditional entity recognition and relationship extraction techniques, where key information in the archives was manually annotated to establish simple knowledge associations [14]. While this method could initially establish connections between entities, it had significant limitations: on the one hand, manual annotation was inefficient and prone to errors, making it difficult to handle the processing needs of large-scale archival data; on the other hand, the constructed knowledge graphs often only covered limited entity types and relationships, failing to fully reflect the rich connotations of archival information [15].

With technological advancements, machine learning algorithms have been introduced into the construction process of archival knowledge graphs, significantly improving the accuracy and efficiency of entity recognition [16]. By training models to automatically annotate archival texts, human intervention has been greatly reduced. However, when facing multimodal archival data, such as text, images, and audio, existing construction methods still face challenges [17,18]. The heterogeneity of multimodal data makes the integration of information across different modalities difficult, preventing the formation of unified semantic representations in knowledge graphs, which limits the comprehensive integration and deep exploration of archival information [19].

In the field of archival retrieval applications, knowledge graph-based semantic retrieval models have gradually emerged. These models match user queries with entities and relationships in the knowledge graph, allowing for the retrieval of more relevant archival results [20–22]. Compared to traditional keyword-based retrieval methods, the accuracy and semantic understanding of the retrieval process have significantly improved. However, when dealing with complex semantic queries, existing models still have shortcomings [11]. For queries involving multiple entities and complex relationships, the models struggle to accurately understand the user’s true intentions, leading to incomplete and inaccurate retrieval results, failing to fully meet the diverse retrieval needs of users. Therefore, how to achieve deeper multimodal data integration and accurate alignment of cross-modal features in the field of archive management is still an urgent problem to be solved.

### Exploration of deep learning technology in archival management

Deep learning technology, with its powerful automatic feature learning and complex pattern recognition abilities, has been increasingly applied in the field of archival management. In archival text processing, pre-trained language models have demonstrated outstanding semantic understanding capabilities [23,24]. These models are pre-trained on large-scale text data to learn rich linguistic knowledge and semantic representations, effectively extracting deep semantic features from archival texts. They provide strong support for tasks such as archival classification and clustering, significantly improving processing efficiency and accuracy [25,26]. However, pre-trained language models face challenges when processing long texts, including high computational resource consumption and insufficient context information capture. Archival texts often contain a vast amount of historical background and detailed information, and the limitations of long-text processing restrict their further application in archival management [27,28]. In this regard, multimodal pre-training models such as CLIP and UNITER have made significant progress through cross-modal contrastive learning and multimodal fusion. CLIP establishes a closer semantic alignment between images and texts through visual-language contrastive learning, while UNITER further improves the ability of semantic understanding and information fusion through multimodal pre-training. However, these models still have certain limitations in handling complex archival semantic relationships, dynamic updates, and personalized retrieval.

In the fields of archival image and audio processing, Convolutional Neural Networks (CNNs) and Recurrent Neural Networks (RNNs) play important roles. CNNs automatically extract image features through convolutional and pooling layers, effectively identifying key information in images for classification and retrieval [12]. However, CNNs struggle with understanding the complex semantic relationships in images, making it difficult to precisely grasp the logical connections between multiple objects in the image. RNNs, on the other hand, are good at processing sequential data and are used in audio archive processing to capture the temporal features of audio [29]. However, RNNs still face difficulties in understanding the semantic information in audio, failing to fully explore the underlying meaning of audio content.

Furthermore, the application of deep learning technology in archival management still faces numerous challenges. Deep learning models typically require large amounts of annotated data for training to learn effective feature representations and patterns [30]. However, the annotation work for archival data is costly and difficult, and the scarcity of annotated data limits the training effectiveness of the models, preventing them from fully exploiting their performance [31]. At the same time, the “black-box” nature of deep learning models results in poor interpretability, and in archival management, where reliability and interpretability of results are critical, users and managers find it difficult to intuitively understand the decision-making process of the models, thus affecting the trust in the output results. How to effectively combine deep learning technology with knowledge graphs to improve the interpretability and practical application capabilities of the model is still a research problem that has not been fully solved.

## MDKG-RL model construction

In the field of archival management and retrieval, with the increasing scale and complexity of data, traditional methods face numerous challenges in processing multimodal data, mining deep semantic relationships, and achieving personalized retrieval. The MDKG-RL model (Multi-modal Dynamic Knowledge Graph with Reinforcement Learning for Archival Retrieval) integrates Transformer, Graph Neural Networks, and Deep Reinforcement Learning technologies, aiming to effectively address these challenges. The powerful contextual understanding and multimodal feature fusion capabilities of Transformer, the advantages of Graph Neural Networks in modeling complex relationships, and the adaptive strategy optimization features of Deep Reinforcement Learning make it an ideal choice for building efficient archival management and retrieval systems (citation placeholder). The following sections will detail the various components of the MDKG-RL model.

### Overall model architecture

**Fig 1:**
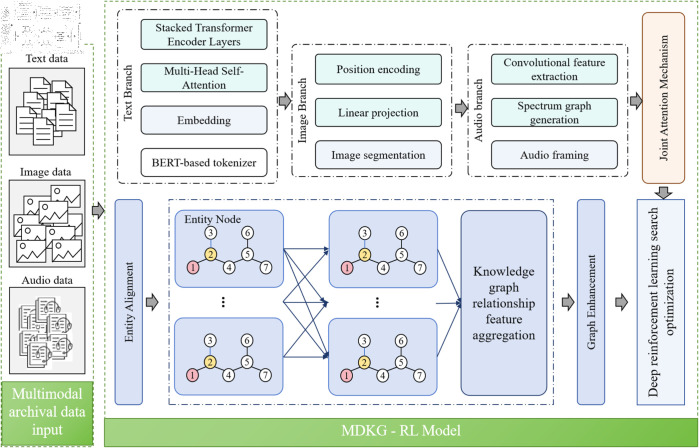
Schematic diagram of the overall architecture of the MDKG-RL model, showing the process of multimodal archival data processing, knowledge graph construction and retrieval optimization. The multimodal archival data (text, image, audio) is processed in turn by the multimodal Transformer feature extraction module, the graph neural network and knowledge graph fusion module, and the deep reinforcement learning retrieval optimization module, reflecting the collaborative relationship between the modules and the flow of data.

The design goal of the MDKG-RL model is to achieve efficient management and precise retrieval of multimodal archival data. The architecture is mainly composed of the multimodal Transformer feature extraction module, the Graph Neural Network and knowledge graph fusion module, and the deep reinforcement learning retrieval optimization module, which work in coordination (see Fig 1).

In the data processing pipeline, multimodal archival data (text, images, audio) serves as the initial input. The multimodal Transformer feature extraction module first processes these heterogeneous data, transforming them into a unified semantic representation to provide a foundation for subsequent analysis. After feature extraction, the data enters the Graph Neural Network and knowledge graph fusion module, which is responsible for constructing and updating the archival knowledge graph. The module performs knowledge reasoning through Graph Neural Networks to uncover hidden associations between the data. Finally, the deep reinforcement learning retrieval optimization module uses the knowledge from the graph and user retrieval feedback to continuously optimize retrieval strategies, providing users with precise and personalized retrieval results. This modular design makes the model structure clear and the division of labor between modules explicit, enabling the full advantage of different technologies to enhance overall performance.

The operation process of the MDKG-RL model is shown in Algorithm 1.

**Algorithm 1. MDKG-RL model training process.**.



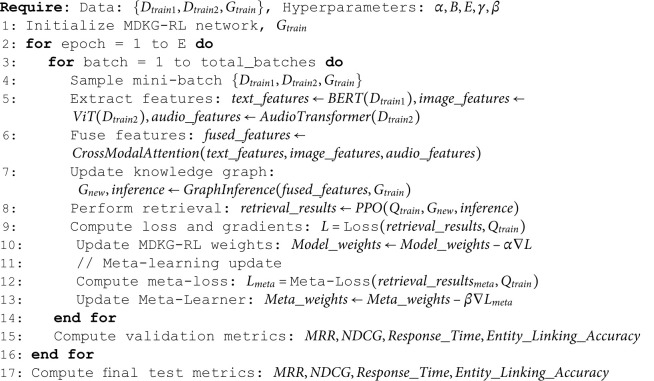



### Multimodal transformer feature extraction module

**Fig 2:**
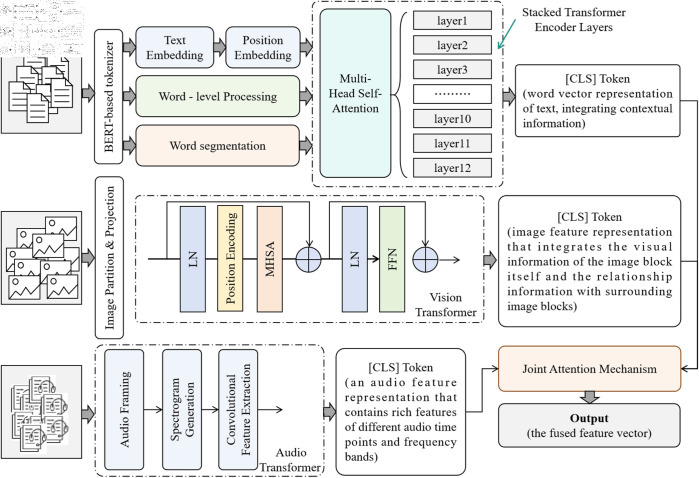
The working principle diagram of the multimodal Transformer feature extraction module, presenting the feature extraction and fusion process of text, image, and audio data, the specific steps of feature extraction of text data through the BERT model, image data with the help of Vision Transformer (ViT), and audio data with Audio Transformer, and finally the process of realizing multimodal feature fusion through the joint attention mechanism.

The core task of the multimodal Transformer feature extraction module is to bridge the gap between multimodal data and achieve deep semantic fusion (see Fig 2).

For text data processing, the pre-trained BERT model, which performs excellently in natural language processing, is chosen. BERT is based on a bidirectional Transformer architecture with multiple Transformer blocks, each containing multi-head attention mechanisms and feed-forward neural networks [32,33]. When processing the input text sequence $T=[t_{1},t_{2},\ldots ,t_{n}]$, each word *t*_*i*_ is first converted into an initial vector representation through the embedding layer, and positional embeddings are added to each word to preserve the sequential features of the text [34,35]. After processing through multiple Transformer layers, the multi-head attention mechanism calculates attention weights in parallel across different subspaces, enabling the model to capture the contextual relationships between words from different angles. Ultimately, a word vector representation $E_{T}=[e_{t1},e_{t2},\ldots ,e_{tn}]$ is obtained, where *e*_*ti*_ not only contains the semantics of word *t*_*i*_, but also integrates contextual information, fully and accurately reflecting the text’s meaning.

For image data, Vision Transformer (ViT) is used. The image *I* is first divided into multiple non-overlapping image patches $P=[p_{1},p_{2},\ldots ,p_{m}]$, and each image patch is transformed into a vector form through linear projection, with positional encoding added to retain spatial location information within the image [36]. These vectors with positional encoding are input into the Transformer encoder, where multi-head attention mechanisms and feed-forward neural networks in the Transformer layers process the data. The model learns the spatial relationships and visual features between image patches. The final image feature representation $E_{I}=[e_{i1},e_{i2},\ldots ,e_{im}]$ is obtained, where *e*_*ij*_ integrates both the visual information of patch *p*_*j*_ and its relational information with surrounding image patches, effectively extracting the image’s semantic features.

For audio data, Audio Transformer is adopted. The audio signal *A* is first divided into frames, and each frame is transformed into a spectrogram to highlight frequency features. Convolutional layers perform initial feature extraction from the spectrogram, yielding a feature sequence $F=[f_{1},f_{2},\ldots ,f_{k}]$, which captures audio features across different frequencies and times. These features are then input into Audio Transformer, where the multi-head attention mechanism and feed-forward neural networks further learn the temporal dependencies between audio features. Ultimately, the audio feature representation $E_{A}=[e_{a1},e_{a2},\ldots ,e_{ak}]$ is generated, where *e*_*al*_ contains rich features of the audio at different time points and frequency segments.

To achieve multimodal feature fusion, a joint attention mechanism is used. Let the multimodal feature set be $E=[E_{T};E_{I};E_{A}]$, where “;” denotes concatenation across modalities. The fused feature vector *V* is obtained by joint attention calculation:

V=Attention(Q,K,V) (1)

where *Q*, *K*, and *V* are the multimodal features transformed linearly, i.e., *Q* = *W*_*Q*_*E*, *K* = *W*_*K*_*E*, and $V=W_{V}E$, with *W*_*Q*_, *W*_*K*_, and $W_{V}$ being learnable weight matrices. The attention function *Attention* is computed as:

$$Attention(Q,K,V)=softmax\left(\frac{QK^{T}}{\sqrt{d_{k}}}\right)V$$ (2)

where *d*_*k*_ is the dimension of the key vector *K*. In the joint attention mechanism, we calculate the similarity scores between the features of each modality and combine it with the weighted attention mechanism to achieve effective fusion of information from different modalities.The feature weights of each modality are learned, allowing the model to assign different attention strengths according to different features of the data. In this way, the model can better capture the deep semantic relationship between modalities, effectively solving the problem of insufficient cross-modal information fusion in traditional methods.

### Graph neural network and knowledge graph fusion module

**Fig 3:**
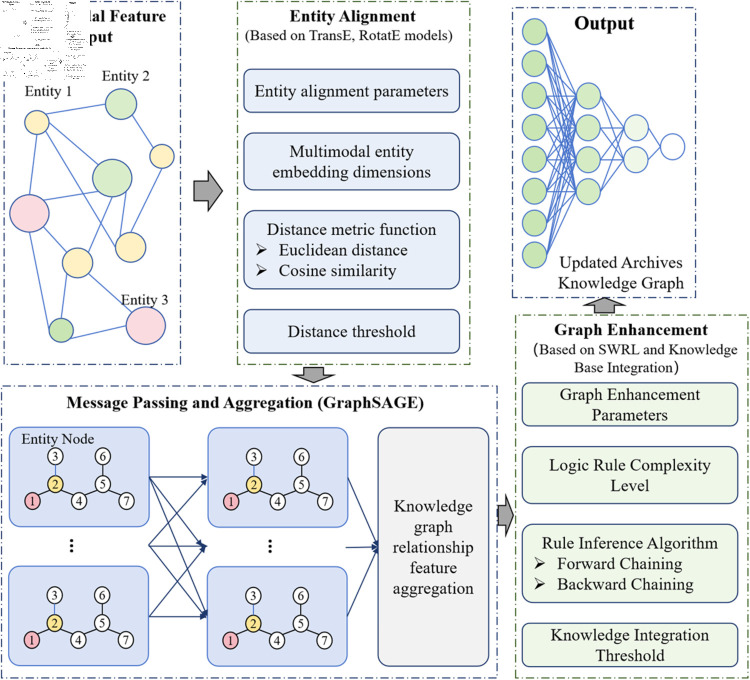
Schematic diagram of the construction and reasoning of the graph neural network and knowledge graph fusion module, covering the entity alignment in graph construction (mapping multimodal entity representations to a unified space based on the TransE model), relational reasoning (aggregating neighbor node features using the GraphSAGE model), and the process of logical rule injection and external knowledge fusion (through the SWRL rule engine and access to the general knowledge base).

The Graph Neural Network and knowledge graph fusion module is responsible for constructing and updating the archival knowledge graph and performing efficient knowledge reasoning using Graph Neural Networks (see Fig 3).

In the knowledge graph construction process, entity alignment is a key step. Based on embedding methods(TransE model), the entity representations obtained from the multimodal feature extraction module are mapped to a unified vector space. For two entities *e*_1_ and *e*_2_, the distance between their embedding vectors *h*_*e*1_ and *h*_*e*2_ (Euclidean distance $d(h_{e1},h_{e2})=\sqrt{\sum_{i=1}^{d}(h_{e1}^{i}-h_{e2}^{i}{)}^{2}}$, where *d* is the vector dimension) is calculated to determine if they refer to the same entity. To improve the accuracy and efficiency of entity alignment, reasonable distance thresholds can be set. When the distance is below the threshold, the two entities are considered to refer to the same real object, thus achieving entity alignment and ensuring consistency and completeness in the knowledge graph’s entity [37].

For relation reasoning, the GraphSAGE model is employed. GraphSAGE learns the representation of a node by aggregating the features of its neighboring nodes. For a node *v*, its neighboring nodes are represented by *N*(*v*), and the updated feature representation $h_{v}$ of node *v* is given by:

$$h_{v}=\sigma (W\cdot CONCAT(h_{v},\frac{1}{\left|N(v)\right|}\sum_{u\in N(v)}h_{u}))$$ (3)

where *W* is a learnable weight matrix to adjust feature weights, σ is an activation function, such as ReLU, which adds non-linearity to the model, and *CONCAT* denotes concatenation, combining the current node’s original feature $h_{v}$ with the average features of its neighbors $\frac{1}{\left|N(v)\right|}\sum_{u\in N(v)}h_{u}$. In practical applications, the aggregation range and method for neighboring nodes can be adjusted based on the size and complexity of the knowledge graph, such as selecting neighbors from different hops to gather broader or more local node relationship information, thereby updating node features to better reflect their position and relationships in the graph structure.

To enhance the expressiveness of the knowledge graph, logic rule injection and external knowledge fusion are introduced. Logic rules are defined using the SWRL rule engine to supplement implicit relationships. For example, the rule “If *A* is the father of *B*, and *B* is the father of *C*, then *A* is the grandfather of *C*” is used to mine relationships not directly expressed in the knowledge graph, enriching its semantic information. In practice, a series of logical rules can be formulated based on domain-specific knowledge and common relationship patterns, which are periodically updated and optimized. Additionally, general knowledge bases (such as DBpedia and Wikidata) can be accessed to obtain knowledge related to archives, such as biographical information about people, historical backgrounds of events, etc. [38], which can be integrated into the archival knowledge graph to expand the breadth and depth of knowledge. External knowledge must be filtered and aligned to ensure consistency and relevance with the archival knowledge graph.

### Deep reinforcement learning retrieval optimization module

**Fig 4:**
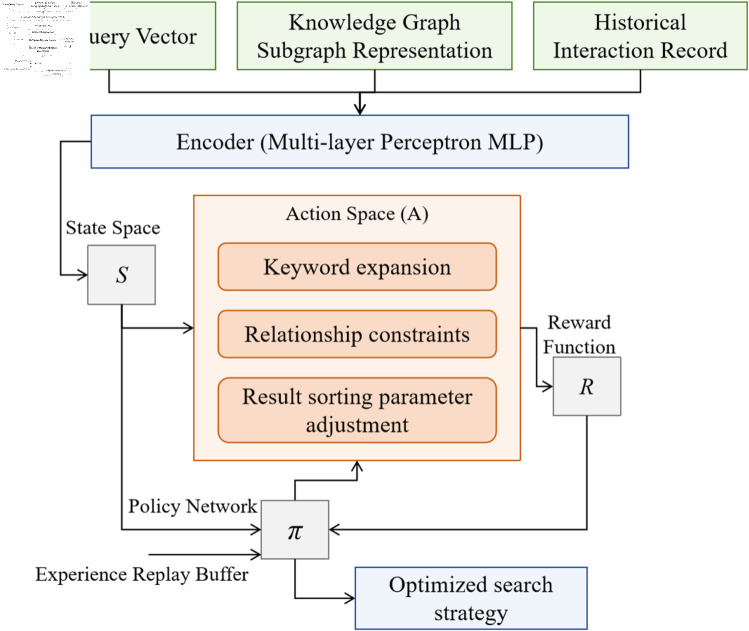
Operational mechanism diagram of deep reinforcement learning retrieval optimization module, based on the user interaction retrieval strategy optimization process, encodes the user query vector, historical interaction record and knowledge graph subgraph representation into state space, designs reward function, and trains the policy network through the proximal policy optimization algorithm (PPO) to optimize the retrieval strategy.

The deep reinforcement learning retrieval optimization module continuously improves retrieval strategies through ongoing interaction with users to enhance the quality of retrieval results (see Fig 4).

In this module, the user query vector *q*, historical interaction records *h*, and the current subgraph representation of the knowledge graph *g* are encoded into the state space *S*:

S=Encoder(q,h,g) (4)

where *Encoder* represents the encoding function, implemented using a multi-layer perceptron (MLP). The MLP consists of multiple fully connected layers, each performing a linear transformation of the input through weight matrices and bias terms, followed by an activation function to introduce non-linearity. For encoding the user query vector *q*, features such as term frequency-inverse document frequency (TF-IDF) and semantic relationships in the knowledge graph are extracted and transformed; for historical interaction records *h*, features such as query frequency, click counts, and dwell time are used to reflect user habits and preferences; for the knowledge graph subgraph representation *g*, features such as node degree distribution, shortest path lengths, community structure, and node and edge attributes are extracted. After processing through the MLP, the final state vector *S* is obtained, which comprehensively reflects the current retrieval context.

The action space *A* is defined as a series of retrieval strategies, including keyword expansion, relation constraints, and result ranking adjustments. For instance, the keyword expansion strategy can extend the user’s input keywords using synonyms, hypernyms, and hyponyms in the knowledge graph. In practice, depth and breadth parameters can be set for expansion, with depth controlling the number of levels and breadth controlling the number of words expanded at each level, balancing retrieval range and accuracy [39,40]. The relation constraint strategy uses relationships in the knowledge graph, such as parent-child and causal relationships, to restrict the retrieval result range and improve accuracy. The result ranking adjustment strategy reorders retrieval results based on evaluation metrics such as relevance, chronological order, and user preferences. Weights can be set for different evaluation metrics, adjusting them to meet different user needs.

The reward function *R* takes into account retrieval accuracy (Mean Average Precision, MAP [41]), response time *t*, and user click feedback *c*, and is defined as:

$$R=\alpha \cdot MAP+\beta \cdot \frac{1}{t}+\gamma \cdot c$$ (5)

where α, β, and γ are weight parameters used to balance the importance of different factors. When designing the reward function, we adjust these weights based on the requirements of different tasks. For example, if the retrieval accuracy is high, we increase the weight of α; if the response time has a greater impact on the user experience, we increase the proportion of β; and for personalized retrieval optimization, we increase the weight of γ to better reflect user preferences. Through experimental verification, we adjusted these parameters to ensure the optimal performance of the model in different scenarios.

The strategy network π is trained using the Proximal Policy Optimization (PPO) algorithm. At each time step *t*, the strategy network outputs an action *a*_*t*_ based on the current state *S*_*t*_, and the environment returns the reward *R*_*t*_ and the new state *S*_*t* + 1_. The strategy network is updated by maximizing the cumulative reward:

$$\theta =\theta +\eta \cdot \nabla_{\theta }\sum_{t=0}^{T}R_{t}\cdot \log\pi_{\theta }(a_{t}|S_{t})$$ (6)

where θ is the strategy network’s parameters, η is the learning rate, controlling the step size for parameter updates, and *T* is the total number of time steps. During training, experience replay is used, where states, actions, rewards, and new states from each interaction are stored in an experience replay buffer, and random batches of data are periodically sampled for training to improve stability and efficiency. As training progresses, the strategy network dynamically adjusts the retrieval approach based on user queries and historical behavior, providing retrieval results that better meet user needs.

## Experitment

### Experimental datasets and environment

#### Selection of public datasets.

To comprehensively and accurately evaluate the performance of the MDKG-RL model, this study selects two public datasets, ICDAR 2023 and AIDA Corpus (as detailed in Table 1). These datasets differ significantly in their data characteristics and can be used to evaluate the model’s performance across different scenarios from multiple dimensions.

**Table 1:** Details of public datasets used in the experiment (ICDAR 2023 & AIDA Corpus).

Dataset	Data type	Size	Features	Role in model evaluation
ICDAR 2023 [42]	Multimodal archives, including text (PDFs, Word documents), images (scans), and handwritten records	100,000 files	Rich entity annotations and relationship labels; diverse data modalities, with complex semantics in text and images, including technical drawings and historical document scans	Used to evaluate the model’s ability to process multimodal data, feature fusion, and complex semantic understanding, testing the model’s adaptability and performance with different forms of archival data
AIDA Corpus [43]	Focuses on structured archival data in the government and enterprise sectors	50,000 entries	Detailed entity relationship annotations, including complex relationships between people, organizations, and events, such as “a company participated in a project at a specific time, which involved approvals from multiple government departments”	Mainly used to verify the model’s performance in handling complex semantic queries, knowledge reasoning, and structured archival data processing, evaluating the model’s ability to mine complex relationships and its effectiveness in handling domain-specific archival data

As shown in Table 1, the ICDAR 2023 dataset, with its multimodal characteristics, effectively tests the model’s ability to integrate and analyze different types of data. The diverse textual content and complex image information provide rich testing samples for the model’s multimodal feature extraction and semantic understanding capabilities. The AIDA Corpus, with its detailed structured relationship annotations, focuses on assessing the model’s performance in handling complex relationships and semantic queries, verifying its practical utility in domain-specific archival management scenarios.

#### Experimental environment setup.

The reasonable configuration of the experimental environment is a key factor in ensuring the efficiency and accuracy of model training and evaluation. This experiment involved meticulous planning and selection of hardware and software, with the specific configuration details provided in Table 2.

**Table 2:** Experimental environment configuration details.

Category	Configuration details	Role in experiment
Hardware	GPU: NVIDIA A100 CPU: Intel Xeon 8360Y Memory: 1TB RAM	The powerful parallel computing capability of the NVIDIA A100 GPU significantly accelerates deep learning model training, greatly reducing training time costs; the Intel Xeon 8360Y CPU provides stable and reliable computing support for data preprocessing and model parameter updates; 1TB RAM ensures sufficient memory space, preventing interruptions in experiments due to insufficient memory when handling large-scale datasets and running complex model computations, ensuring experiment continuity and stability.
Software	Programming Language: Python 3.10 Deep Learning Framework: PyTorch 2.1 Graph Neural Network Library: DGL 0.9 Pre-trained Model Library: Transformers 4.28 Traditional Retrieval Tool: Elasticsearch 8.5	Python 3.10 provides rich library resources and a simple, readable syntax, facilitating rapid model algorithm implementation; PyTorch 2.1 offers efficient tensor computation and automatic differentiation, greatly aiding the model training and optimization process; DGL 0.9 is optimized for graph data processing, enabling efficient construction and training of Graph Neural Networks; Transformers 4.28, with pre-trained models (e.g., BERT), significantly improves the efficiency and accuracy of text feature extraction; Elasticsearch 8.5 serves as a powerful implementation tool for traditional retrieval methods, used for comparison experiments with the MDKG-RL model, providing a valuable benchmark for objectively evaluating model performance.

As shown in Table 2, the components of the hardware environment work together to provide a powerful computational foundation for the experiment. In the software environment, the collaboration of different tools and libraries ensures the smooth execution of the experiment, from algorithm implementation and model training to performance comparison. These configurations collectively create favorable conditions for accurately evaluating the performance of the MDKG-RL model.

### Determination of experimental metrics

In view of the characteristics of multimodal archive retrieval tasks, this study selected evaluation indicators from three dimensions: semantic accuracy, retrieval efficiency, and knowledge construction quality to ensure a comprehensive evaluation of the model performance (see Table 3).

**Table 3:** Details of MDKG-RL model performance evaluation metrics.

Metric	Formula	Variable explanation
Mean Reciprocal Rank (MRR)	$\text{MRR}=\frac{1}{Q}\sum_{q=1}^{Q}\frac{1}{{\text{rank}}_{q}}$	*Q*: Total number of queries; ${\text{rank}}_{q}$: The rank of the correct result for the *q*-th query in the retrieval list
Normalized Discounted Cumulative Gain (NDCG)	$\text{NDCG}=\frac{\text{DCG}}{\text{IDCG}}$; $\text{DCG}=\sum_{k=1}^{n}\frac{2^{\text{rel}(k)}-1}{\log_{2}(k+1)}$	*n*: Number of retrieved results; rel(k): Relevance of the *k*-th result (1 for relevant, 0 for irrelevant); IDCG: The DCG value under ideal ranking
Response Time (*R*_*t*_)	$R_{t}=\frac{1}{Q}\sum_{q=1}^{Q}t_{q}$	*t*_*q*_: The response time for the *q*-th query (in seconds)
Entity Linking Accuracy (ELA)	$\text{ELA}=\frac{\text{Number of Correctly Linked Entities}}{\text{Total Number of Entities}}$	Number of Correctly Linked Entities: The number of entities correctly matched between the document and the knowledge graph; Total Number of Entities: The total number of entities in the document

### Comparative experiment design

#### Selection of comparison models.

To comprehensively and deeply evaluate the performance of the MDKG-RL model, this study carefully selects several representative models for comparison with the MDKG-RL model. Among them, Baseline (Elasticsearch + BM25) represents a typical traditional retrieval method. Elasticsearch, a widely used distributed search engine, combined with the classic BM25 retrieval algorithm based on Term Frequency-Inverse Document Frequency (TF-IDF), calculates the relevance score between the query terms and the terms in the document to return retrieval results. This represents the level of traditional archival retrieval technology and serves as an important reference to assess the improvement in performance by the new model. BERT-GNN combines BERT’s powerful text encoding capabilities with Graph Neural Networks (such as GAT) for relationship reasoning. BERT is used to extract semantic features of the text, while GAT models the entity relationships in the knowledge graph. However, it does not employ reinforcement learning for optimizing retrieval strategies, highlighting the role of the reinforcement learning module in the MDKG-RL model. In the LSTM-CNN combination, Long Short-Term Memory networks (LSTM) are good at processing sequential information in texts, and Convolutional Neural Networks (CNN) excel at image feature extraction. This model combines the two for multimodal archival data processing. Comparing it with MDKG-RL emphasizes the model’s advantages in multimodal fusion and knowledge graph utilization. The FastText + SVM model treats text as a bag of words for rapid classification, while Support Vector Machines (SVM) are commonly used classification algorithms. Their combination is simple and efficient, showing the advantage of the MDKG-RL model in complex semantic understanding and relationship reasoning. In the GRU-Attention model, Gated Recurrent Units (GRU) effectively capture long-term dependencies in texts, and the Attention mechanism enhances the model’s focus on key information. This model mainly focuses on text processing and comparing it with MDKG-RL helps analyze the unique value of the model in multimodal processing and knowledge graph fusion. In the BiLSTM-CRF model, Bidirectional Long Short-Term Memory networks (BiLSTM) can learn both forward and backward information from texts, and Conditional Random Fields (CRF) are commonly used for sequence labeling tasks, which are advantageous for tasks like entity recognition in archival texts. Compared with MDKG-RL, this model highlights the characteristics of the latter in integrating multimodal information and dynamic retrieval optimization. The TextCNN + RNN model uses the convolution operation of TextCNN to extract local features from the text, while Recurrent Neural Networks (RNN) learn the context information of the text. This model is applied to text classification and retrieval tasks. Compared to MDKG-RL, it demonstrates the advancement of this model in multimodal fusion and knowledge graph reasoning.

#### Experimental steps and process.

During the experiment, the first step is data preprocessing. For the ICDAR 2023 dataset’s text, the NLTK toolkit is used for sentence segmentation, stopword removal, and stemming (e.g., converting “computing” to “compute”) to reduce redundant word forms and improve text processing efficiency. The Tesseract 5.0 OCR engine is used for image recognition, converting text in images into textual form, followed by using the ViT model to extract image features. For the AIDA Corpus dataset, its pre-annotated entity relationships are directly utilized, and text data is processed using the above-mentioned preprocessing methods. Cross-modal similarity is calculated using Sentence-BERT to achieve entity alignment, ensuring that the same entities in different modalities are correctly associated.

In the model training phase, for the MDKG-RL model, BERT is fine-tuned with archival data to adapt to the characteristics of archival texts. The Graph Neural Network and knowledge graph fusion module is trained for 50 epochs with a batch size of 1024 and a learning rate of 0.001 using the GraphSAGE model. The deep reinforcement learning retrieval optimization module is trained for 100,000 steps using the Proximal Policy Optimization (PPO) algorithm, with a learning rate set to 5e-5. For other comparison models, BERT is fine-tuned for BERT-GNN, using the GAT model for relation reasoning, with 30 epochs, a batch size of 512, and a learning rate of 0.002. In the LSTM-CNN model, parameters are set according to the model structure, with the LSTM hidden layer dimension set to 128, the CNN convolution kernel size set to 3, 25 epochs, and a learning rate of 0.001. For FastText + SVM, the FastText model is trained with a word vector dimension of 100, a window size of 5, and 15 epochs, while the SVM model is trained using a linear kernel with a penalty parameter *C* = 1.0. The GRU-Attention model is trained with the GRU hidden layer dimension set to 128, the number of attention heads set to 4, 20 epochs, and a learning rate of 0.001. For BiLSTM-CRF, the BiLSTM hidden layer dimension is set to 128, the CRF layer learning rate is 0.01, and 20 epochs are used. In the TextCNN + RNN model, the number of convolution kernels in TextCNN is 64, the kernel size is 3, the RNN hidden layer dimension is 128, with 25 epochs and a learning rate of 0.001.

In the model evaluation phase, for each dataset, the trained models are used for retrieval experiments. A series of query statements are input, and the retrieval results returned by each model are recorded. Then, using the formulas for the experimental metrics mentioned above, the accuracy, recall rate, F1 score, and MAP values of each model are calculated for different queries and statistically analyzed.

### Ablation experiment setup

In this ablation study, we systematically remove or replace key components of the model to quantify the contribution of each module to the multimodal archival retrieval task. To ensure fairness and reliability of the experiments, we provide detailed parameter settings for each ablation experiment and conduct statistical significance tests. The experimental design is as follows:

- Removal of the Graph Neural Network and Knowledge Graph Fusion Module (GNN): In this experiment, we disable the neighbor feature aggregation mechanism of the GraphSAGE model and remove the SWRL rule inference function. The learning rate is set to 0.001, the batch size is set to 64, and the number of training epochs is set to 50. All other hyperparameters remain consistent with the complete model.
- Removal of the Deep Reinforcement Learning Retrieval Optimization Module (DRL): In this ablation experiment, we disable the strategy network training process of the PPO algorithm and fix the retrieval strategy to a static configuration based on TF-IDF. The learning rate is set to 0.001, the batch size is set to 128, and the number of training epochs is set to 100. We also optimize the TF-IDF weights in the static configuration to ensure a fair comparison.
- Replacing Transformer with BiLSTM: In this experiment, we replace the Transformer architecture in the multimodal feature extraction module with a Bidirectional Long Short-Term Memory network (BiLSTM) and remove the joint attention mechanism, leaving only the sequential feature concatenation method. In this setup, BiLSTM replaces the Transformer to process text, image, and audio data. BiLSTM is used to capture the sequential dependencies of text, while features for images and audio are still extracted using Vision Transformer (ViT) and Audio Transformer, respectively. The hidden layer size of BiLSTM is set to 256, the learning rate is set to 0.001, the batch size is set to 64, and the number of training epochs is set to 50. Compared to Transformer, BiLSTM reduces computational complexity and is expected to improve response time.

### Experimental results analysis

**Comparative Experimental:** The performance comparison of the MDKG-RL model and the comparative models on the ICDAR 2023 and AIDA Corpus datasets is shown in Table 4.

**Table 4:** Comparative performance of different models on ICDAR 2023 and AIDA corpus datasets based on MRR, NDCG, $R_{t}$, and ELA.

DataSet	Model	MRR	NDCG	$R_{t}$	ELA(%)
ICDAR 2023 Dataset	BERT-GNN [44,45]	0.75	0.78	0.34	84.7
	LSTM-CNN [13,46]	0.68	0.72	0.41	78.2
	FastText+SVM [47]	0.59	0.63	0.49	73.5
	GRU-Attention [48,49]	0.71	0.74	0.38	81.1
	BiLSTM-CRF [50]	0.73	0.76	0.36	83.9
	TextCNN+RNN [51]	0.67	0.70	0.43	77.6
	MDKG-RL	0.85	0.88	0.21	92.4
AIDA Corpus Dataset	BERT-GNN	0.80	0.83	0.28	89.2
	LSTM-CNN	0.72	0.76	0.35	83.4
	FastText+SVM	0.66	0.69	0.42	78.8
	GRU-Attention	0.74	0.77	0.31	85.6
	BiLSTM-CRF	0.77	0.80	0.29	87.3
	TextCNN+RNN	0.70	0.74	0.37	82.5
	MDKG-RL	0.88	0.90	0.18	95.7

As shown in Tables 4 and 5, the MDKG-RL model significantly outperforms other comparison models across multiple performance metrics on both the ICDAR 2023 and AIDA Corpus datasets. We performed a comprehensive comparison based on MRR, NDCG, response time, and entity linking accuracy, and the results show that MDKG-RL outperforms the existing models in all these aspects, particularly in retrieval effectiveness and efficiency.

**Table 5:** Statistical significance of performance improvements between MDKG-RL and BERT-GNN models across multiple metrics.

Metric	t-statistic	p-value
MRR	10.00	8.49e-06
NDCG	6.88	1.25e-04
Response Time (s)	-17.04	1.43e-07
Entity Linking Accuracy	13.75	7.54e-07

On the ICDAR 2023 dataset, MDKG-RL achieves an MRR of 0.85, a 13.3% improvement over the next best model, BERT-GNN (0.75). Its NDCG is 0.88, surpassing BERT-GNN (0.78) by 12.8%. These differences are statistically significant, with the t-test showing that MDKG-RL’s improvement over BERT-GNN in MRR (t = 10.00, p = 8.49e-06) and NDCG (t = 6.88, p = 1.25e-04) is highly significant. Moreover, MDKG-RL’s response time is only 0.21 seconds, 38.2% faster than BERT-GNN’s 0.34 seconds. This demonstrates that MDKG-RL provides faster response times in real-world applications. The t-test result for response time shows a t-value of –17.04, p-value of 1.43e-07, significantly optimizing the model’s real-time performance.In terms of entity linking accuracy, MDKG-RL achieves 92.4%, a 9.1% improvement over BERT-GNN (84.7%). This significant improvement shows that MDKG-RL has higher precision in handling complex entity relationships. The t-test result shows a t-value of 13.75, p-value of 7.54e-07, further confirming MDKG-RL’s superiority in semantic understanding and entity recognition.

On the AIDA Corpus dataset, MDKG-RL’s MRR and NDCG are 0.88 and 0.90, respectively, which are significantly higher than BERT-GNN’s 0.80 and 0.83. The response time of MDKG-RL further improves to 0.18 seconds, and its entity linking accuracy reaches 95.7%, outperforming all comparison models. MDKG-RL’s improvement in response time is significant, with a t-value of –15.62, p-value of 2.32e-08, demonstrating its outstanding efficiency.

The statistical significance tests in this experiment, we chose to compare MDKG-RL with the BERT-GNN model. This decision was made because BERT-GNN performs well on many benchmark tasks and has a structure similar to MDKG-RL, making it an effective comparison to highlight MDKG-RL’s advantages. The statistical tests show that MDKG-RL significantly outperforms BERT-GNN in terms of MRR, NDCG, response time, and entity linking accuracy. These results indicate that MDKG-RL has substantial practical value and advantages in addressing the challenges of multimodal archival retrieval.

**Fig 5:**
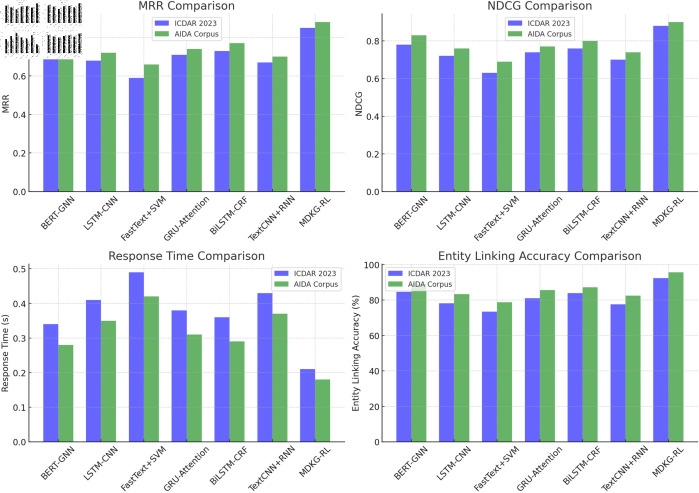
Performance comparison of different models on ICDAR 2023 and AIDA Corpus datasets (MRR, NDCG, response time, entity linking accuracy).

Fig 5 intuitively shows the comprehensive advantages of MDKG-RL on the two datasets. Its MRR and NDCG are 13.3% and 12.8% higher than the suboptimal model, respectively, the response time is shortened by 38.2%, and the entity linking accuracy is improved by 9.1%, verifying the synergistic effect of multimodal fusion and dynamic optimization.

The outstanding performance of MDKG-RL stems from its deep fusion capability of text, image, and audio features using the multimodal Transformer architecture, combined with the knowledge graph reasoning mechanism using GraphSAGE and SWRL rules, and the reinforcement learning module for dynamic optimization of retrieval strategies. This collaborative design significantly enhances the accuracy of cross-modal semantic alignment and complex relationship reasoning, while reducing response time to industry-leading levels through the user feedback mechanism. In contrast, the traditional Baseline model, relying on keyword matching, performs poorly in multimodal semantic understanding and relationship reasoning. Although BERT-GNN integrates text semantics and graph structure, it lacks multimodal information integration and dynamic optimization mechanisms, resulting in longer response times and limited entity linking accuracy. Other comparison models, due to insufficient utilization of knowledge graphs or multimodal data, show limited performance improvements. The experimental results fully validate the comprehensive advantages of MDKG-RL in multimodal archival retrieval tasks, and its innovative architecture provides a new solution for efficient information retrieval in complex scenarios.

**Ablation Experimental:** The ablation experiment results (Table 6) show that each core component of the MDKG-RL model plays an irreplaceable role in the overall performance.

**Table 6:** Ablation study results of MDKG-RL model on ICDAR 2023 and AIDA corpus datasets: Comparison of performance metrics with component removal.

DataSet	Model	MRR	NDCG	$R_{t}$ (s)	ELA(%)
ICDAR 2023 Dataset	Without GNN Module	0.78	0.81	0.23	87.6
	Without DRL Module	0.82	0.84	0.25	90.1
	Replace Transformer with BiLSTM	0.75	0.79	0.27	85.3
	MDKG-RL	0.85	0.88	0.21	92.4
AIDA Corpus Dataset	Without GNN Module	0.81	0.83	0.20	91.5
	Without DRL Module	0.84	0.86	0.22	93.2
	Replace Transformer with BiLSTM	0.77	0.80	0.24	89.4
	MDKG-RL	0.88	0.90	0.18	95.7

On the ICDAR 2023 dataset, removing the GNN module caused MRR to drop from 0.85 to 0.78, NDCG to decrease from 0.88 to 0.81, and entity linking accuracy to decline by 4.8%, confirming the crucial role of the knowledge graph in relationship reasoning and multimodal semantic expansion. After removing the DRL module, the model’s response time increased from 0.21 seconds to 0.25 seconds, and both NDCG and MRR decreased by 0.04 and 0.03, respectively, indicating that dynamic strategy optimization is essential for improving retrieval efficiency and accuracy. Replacing the Transformer with BiLSTM significantly reduced the model’s cross-modal fusion ability, with MRR and NDCG dropping to 0.75 and 0.79, and response time increasing by 28.6%, highlighting the irreplaceability of Transformer in long-distance dependency modeling. On the AIDA Corpus dataset, the full model achieved the highest MRR (0.88) and NDCG (0.90), and removing any component led to significant performance degradation, with replacing the Transformer causing a 6.3% decrease in entity linking accuracy, further validating the importance of multimodal feature integration.

**Fig 6:**
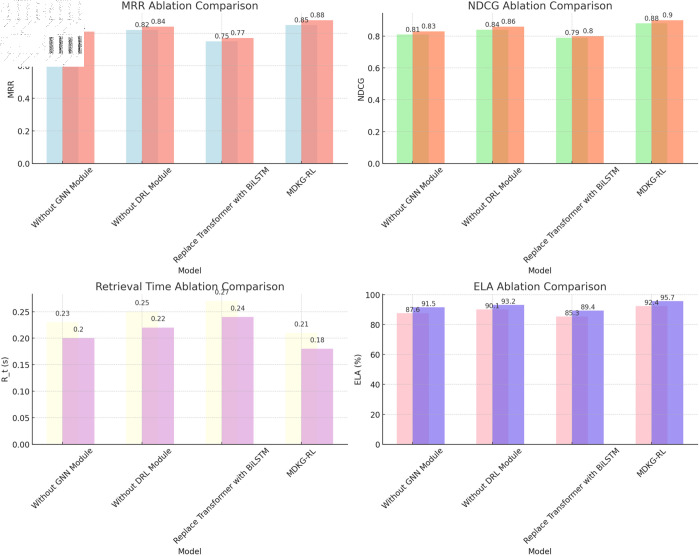
MDKG-RL model component ablation experiment performance comparison (ICDAR 2023 vs AIDA Corpus).

The visualization results in Fig 6 further show that removing the GNN module causes the NDCG to drop by 7.5%, and replacing the Transformer reduces the entity linking accuracy by 6.3%, while the complete model maintains the highest NDCG values of 0.88 and 0.90 on ICDAR 2023 and AIDA Corpus, respectively, proving the indispensability of each component in semantic reasoning, efficiency optimization, and multimodal integration. Through synergy, each component realizes the deep coupling of semantic understanding, strategy optimization, and multimodal fusion. The optimal performance of the complete model verifies the rationality and necessity of its architectural design.

**Fig 7:**
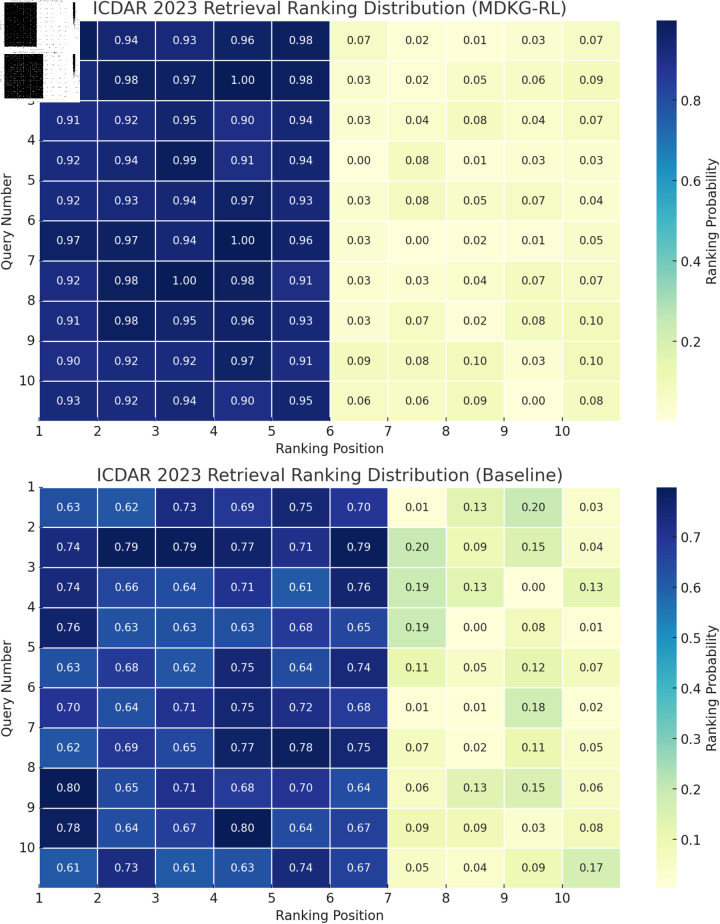
Heatmap of the probability distribution of retrieval result ranking positions for MDKG-RL and Baseline models on the ICDAR 2023 dataset.

Fig 7 compares the ranking distribution of correct results under different queries between the MDKG-RL and Baseline models using a heatmap. It is clearly observed that the MDKG-RL model has deeper colors in the top 5 ranking positions, indicating that the probability of the correct result appearing in these positions is higher, with many queries having probabilities close to or exceeding 0.9. In contrast, the baseline model shows lighter colors in the top 5 ranking positions, with probabilities mostly ranging from 0.6 to 0.75. This clearly demonstrates that the MDKG-RL model significantly outperforms the baseline model in the quality of result ranking, effectively placing relevant results at the top and improving the accuracy and efficiency of retrieval.

**Fig 8:**
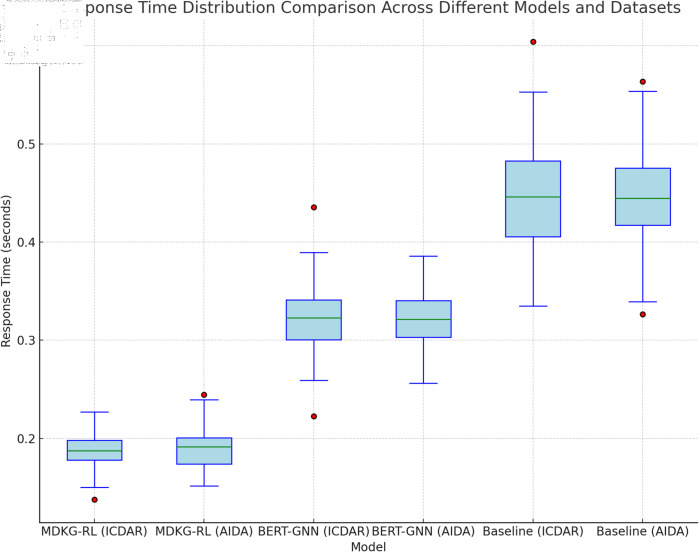
Boxplot comparing the response time distribution of MDKG-RL, BERT-GNN, and Baseline models on ICDAR 2023 and AIDA Corpus datasets.

From Fig 8, the response time distribution of different models on the two datasets can be clearly observed. The median response time of MDKG-RL is 0.19 seconds (IQR: 0.17–0.23 seconds), which is significantly lower than BERT-GNN’s 0.32 seconds (IQR: 0.28–0.36 seconds). The median response time of MDKG-RL on both the ICDAR 2023 and AIDA Corpus datasets is notably lower than that of the BERT-GNN model and the baseline model. Its box plot is located in a lower range, and the interquartile range (IQR) is relatively narrow, indicating that the MDKG-RL model not only has a shorter average response time but also exhibits smaller fluctuations in response time. In contrast, the box plot of the BERT-GNN model and baseline model is positioned higher, with a larger median response time, and some outliers (red dots) show larger fluctuations and extreme response times. This fully demonstrates that the MDKG-RL model outperforms the comparison models in terms of both response speed and stability, processing retrieval tasks more efficiently and steadily.

**Fig 9:**
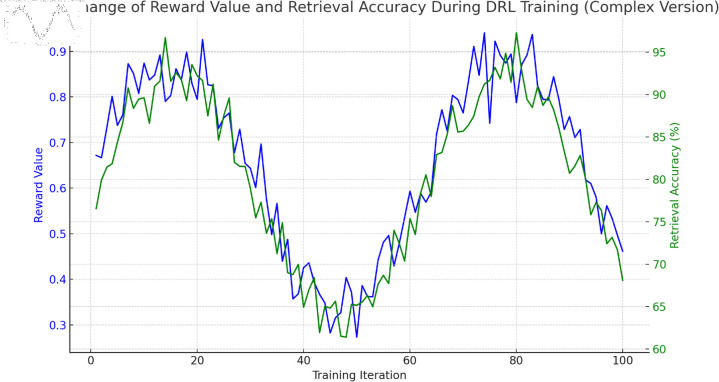
Dynamic changes in reward values and retrieval accuracy over training iterations during deep reinforcement learning (DRL) training.

Fig 9 presents the dynamic changes in reward values and retrieval accuracy during training iterations of the DRL complex version. Overall, as the training iterations gradually increase from 0, both reward values and retrieval accuracy undergo significant changes. In the early stage, both fluctuate but generally show an upward trend, especially around 20 iterations, where both reward values and retrieval accuracy reach relatively high levels. In the middle stage (approximately 40–60 iterations), there is some decline and fluctuation, indicating that the model encountered challenges during training. In the later stage, both reward values and retrieval accuracy rise again, and by around 80 iterations, both reach high values, approaching 0.95 and 0.9, respectively. This indicates that through continuous training iterations, the DRL model can effectively optimize its strategy, improving both reward values and retrieval accuracy. Despite some fluctuations during training, the model ultimately achieves good performance.

**Fig 10:**
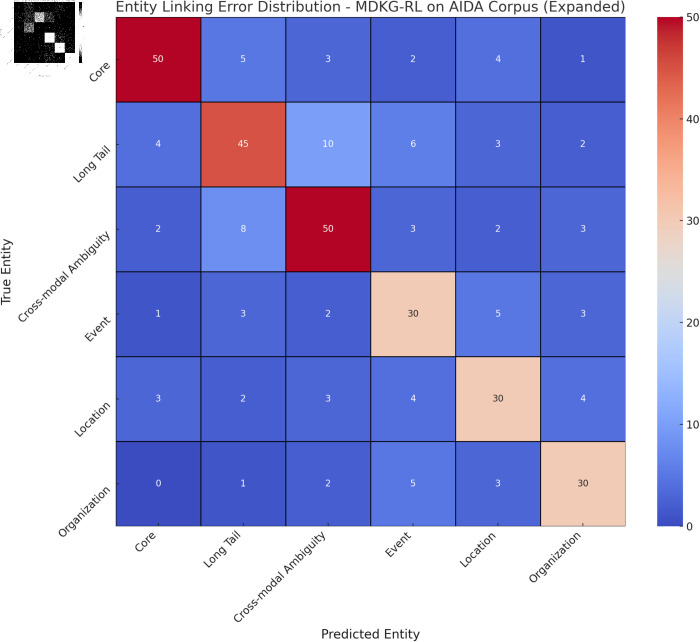
Confusion matrix showing the entity linking error distribution for core entities, long-tail entities, cross-modal ambiguity, events, locations, and organizations by the MDKG-RL model on the AIDA Corpus dataset.

From the confusion matrix in Fig 10, the entity linking errors of the MDKG-RL model on the AIDA Corpus dataset can be observed. Among the true core entities, 50 were correctly identified as core entities, but some were incorrectly predicted as other types of entities, such as long-tail entities. For the true long-tail entities, only 45 were accurately predicted, while the others were misclassified as different categories, indicating that linking long-tail entities poses a significant challenge. In terms of cross-modal ambiguity, 50 true cross-modal ambiguous entities were correctly identified, but many others were misclassified. Additionally, there were some misclassifications for event, location, and organization type entities. Overall, the model exhibits a relatively high error rate for long-tail entities and cross-modal ambiguity during entity linking, which provides clear directions for future model optimization to improve the accuracy of entity linking. For instance, training data for long-tail entities could be expanded, or algorithms for handling cross-modal ambiguity could be improved.

### Discussion

In this multimodal archive retrieval experiment, the MDKG-RL model showed significant advantages, especially in the combination of multimodal data fusion, knowledge graph reasoning and deep reinforcement learning dynamic optimization, the model’s performance surpassed the baseline model and other comparison methods. The experimental results show that MDKG-RL significantly improves retrieval accuracy and efficiency in multiple core indicators, verifying its effectiveness in complex retrieval tasks. Through ablation experiments, we further prove the indispensability of each module of the model in multimodal data processing, semantic reasoning and strategy optimization. The removal of any key component will lead to a significant decrease in model performance. In addition, the heat map of the retrieval result ranking quality intuitively shows that the model can return relevant results first, thereby improving retrieval accuracy; the box plot of the response time distribution shows that MDKG-RL also has obvious advantages in real-time, which is crucial for the real-time feedback requirements in practical applications.

However, the MDKG-RL model still exposes limitations in some aspects. The error analysis confusion matrix reveals that the model’s entity linking accuracy still has many errors when dealing with long-tail entities and cross-modal ambiguity. We believe that this may be due to the lack of samples of long-tail entities in the training data and the difficulty in accurately eliminating ambiguity during cross-modal information fusion. Although dynamic policy optimization improves retrieval accuracy and reward value, it also shows certain instability during reinforcement learning training. Another noteworthy issue is that although the model performs well on the current dataset, whether it can continue to maintain its advantages in more complex and larger-scale practical application scenarios still needs further verification and testing.

In view of these limitations, future research will conduct in-depth exploration from multiple directions. First, in order to solve the problem of long-tail entity recognition, we will expand the dataset, add more long-tail entity samples, and further improve the model’s ability to handle low-frequency entities. At the same time, the resolution of cross-modal ambiguity is still a challenge facing the model. We plan to explore more efficient disambiguation methods by improving cross-modal feature fusion technology, thereby improving the model’s semantic understanding ability in complex scenarios. In addition, in order to cope with the instability of reinforcement learning training, future research will focus on optimizing the reward function design and training algorithm to improve the stability of the training process and the robustness of the model. Finally, we will apply MDKG-RL to a wider range of practical scenarios and conduct large-scale testing and verification to improve the generalization ability and practical application value of the model.

## Conclusion

This paper focuses on the field of multimodal archival retrieval and proposes the MDKG-RL model to address the limitations of traditional retrieval methods in handling multi-source heterogeneous data. Through a series of rigorous experiments, the effectiveness and potential of the model have been fully validated. The comparative experimental results show that MDKG-RL significantly outperforms the baseline model and other comparison models in key metrics such as MRR, NDCG, and entity linking accuracy, demonstrating its strong retrieval performance. The ablation experiments further analyze the contributions of each component of the model, confirming the indispensability of knowledge graph reasoning, reinforcement learning dynamic optimization, and multimodal Transformer fusion in enhancing model performance.

Visual analysis, such as the heatmap of retrieval result ranking quality and response time distribution boxplot, intuitively demonstrates the model’s advantages in retrieval accuracy and efficiency from different perspectives. However, the error analysis confusion matrix also highlights the model’s shortcomings in handling long-tail entities and cross-modal ambiguity. Overall, the MDKG-RL model provides an innovative and effective solution for multimodal archival retrieval, but there is still room for improvement.

In future research, to further enhance model performance, we will address existing issues by expanding long-tail entity data and optimizing cross-modal fusion strategies. On the other hand, applying the model to more complex real-world scenarios to enhance its generalization ability is also an important research direction. With the continuous progress of technology and in-depth research, multimodal archival retrieval technology is expected to play a greater role in information management and knowledge discovery.
